# Poor mental health days is associated with higher odds of poor oral health outcomes in the BRFSS 2020

**DOI:** 10.1186/s12903-022-02543-1

**Published:** 2022-11-16

**Authors:** Hoda M. Abdellatif

**Affiliations:** Department of Preventive Dental Sciences, College of Dentistry, Community Division, Princess Nourah Bint Abdulrahman University, PO Box 84428, Riyadh, Saudi Arabia

**Keywords:** Mental health, Oral health, Behavioral Risk Factor Surveillance System, Chronic disease, Healthcare disparities, United States

## Abstract

**Background:**

To test the hypothesis that among individuals in the 2020 Behavioral Risk Factor Surveillance System (BRFSS) cross-sectional anonymous health survey in the United States (US), after controlling for confounding, an increasing number of poor mental health (MH) days in the past month is associated with increasing odds of delayed oral health (OH) care utilization and poorer OH outcomes.

**Methods:**

Adjusted logistic regression models were developed with poor MH days as the exposure to examine the association with two dependent variables (DVs): Most recent dental visit longer than one year ago (yes/no), and having lost 6 or more teeth (yes/no).

**Results:**

Approximately one third (32%) reported most recent dental visit more than one year ago, and 17% had lost 6 or more teeth. Those in the second quartile of poor MH days had 11% higher odds of delayed dental visit, and those in the highest quartile had 26% higher odds, compared to the reference group. For having lost 6 or more teeth, compared to the reference group, those in the third quartile had 8% higher odds and those in the fourth quartile had 18% higher odds.

**Conclusions:**

Poor MH days is independently associated with odds of poor OH utilization and OH in the US above and beyond diagnosed mental and physical conditions. Policymakers in the US should expand health insurance plans to include dental insurance, and should increase access to MH care, especially for the aging population, and those with chronic conditions.

## Background

The United States (US) healthcare system is recognized as being one of the most expensive in the world, while having extreme barriers to access and producing some of the worst outcomes [1, 2]. Specifically, those in the US who need mental health (MH) care often encounter barriers to access, limited availability, and low quality treatment [3, 4]. A population-based study of over 50,000 US adults found that 95.6% reported at least one barrier to healthcare access, 13.3% had no usual source of care, and those with MH challenges were more likely to report access barriers [3]. Also, in the US, the oral healthcare system is completely separate from the rest of the healthcare system, such that most health insurances (including the public insurances Medicare and Medicaid) do not provide dental coverage, leading to severe barriers in access to oral healthcare, especially among the poor [5]. While there may be personal reasons to avoid oral healthcare, such as fear of pain, in the US, the lack of access due to financial or other reasons presents the largest barrier [6]. In a study estimating population-level rates in the US in 2011–2014, authors found that the prevalence of untreated caries was 15.9% for children and 25.0% for adults, with a large proportion reporting both financial and non-financial barriers [6].

### Longitudinal relationship of poor mental health to poor oral health

This circumstance implies that in the US population, there may be a subpopulation of individuals who need but lack access to care for physical, MH, and oral health (OH) needs, so it would be helpful to know the mutual influence of these risk factors on each other. The direction and mechanism behind the causative associations between poor MH and poor OH have historically been elusive, but recent large epidemiologic studies have shed light on the subject [7–10]. Much of the challenge lies in the diversity of classification of “poor MH” as a risk factor across epidemiologic studies, where misclassification may easily occur. First of all, “poor MH” can be defined as carrying a diagnosed mental disorder, such as bipolar disorder or depression, or a clinically-diagnosed neurological condition that impacts behavior such as dementia [10], that can be assessed clinically or self-reported. Patients who fall in such a classification represent a biased group, in that they are more likely to have achieved access to healthcare so as to have been able to obtain a diagnosis, and their MH condition is likely more severe [11, 12].

Secondly, “poor MH” could be defined as a self-reported measure of poor MH as a proxy for the state of health-related quality-of-life (HRQOL) [13, 14]. In the cross-sectional Behavioral Risk Factor Surveillance System (BRFSS) annual phone survey completed nationally by the US government, in addition to asking about clinically-diagnosed depression, the survey asks, “Now thinking about your mental health, which includes stress, depression, and problems with emotions, for how many days during the past 30 days was your mental health not good?”. Although the question is used to estimate a particular 30-day window, this question has long been considered a reliable and valid measure of HRQOL [14].

This HRQOL measurement of poor MH days, rather than a diagnosable condition, measures a construct, similar to the measurement of social isolation and/or loneliness [8]. An study hypothesizing that social isolation and loneliness lead to poor OH outcomes was done using three waves of the Chinese Longitudinal Healthy Longevity Survey (CLHLS) data to assess the impact of social isolation and loneliness on incident tooth loss [8]. In this study, social isolation was defined using four criteria, and loneliness was measured using one Likert-scale item [8]. As the dependent variable used in regression models in this study was “number of remaining teeth”, although their results clearly demonstrated that higher levels of social isolation and loneliness at baseline were statistically significantly associated with accelerated tooth loss in subsequent years, it is difficult to interpret their numerical results clinically [8].

### Cross-sectional associations between poor mental health and poor oral health

A cross-sectional analysis of data from the English Longitudinal Study of Ageing (ELSA) found that after controlling for confounding, the odds of loneliness were 1.48 higher among those with at least one OH impact on daily life (compared to no impacts), and found that increasing loneliness was associated with increasing odds of additional OH impacts [9]. In this study, loneliness was measured through three Likert scale items, and oral health impacts were measured using the Oral Impacts on Daily Performances (OIDP) scale [9]. In a meta-analysis of 14 cross-sectional studies assessing the association between poor MH and poor OH, 11 considered MH as the risk factor for poor OH, and only three implied a reversed causal direction [7]. It is important to note that in epidemiologic studies like these where measurement of a disease condition is obtained using a questionnaire-based survey, the risk is higher of false-positive and false-negative responses from the participants in terms of misclassification of disease. Further, the structure of these questions limits the analysis; in the cross-sectional BRFSS annual survey in the US, number of lost teeth is classified into the categories “none”, “one to five”, “six or more”, and “all” [15]. Questionnaires also seek to measure non-clinical concepts such as “loneliness” and “poor MH days”, and this can also be a source of measurement error [8, 15].

### Theoretical disease progression model of poor mental health and poor oral health

**Fig. 1:**
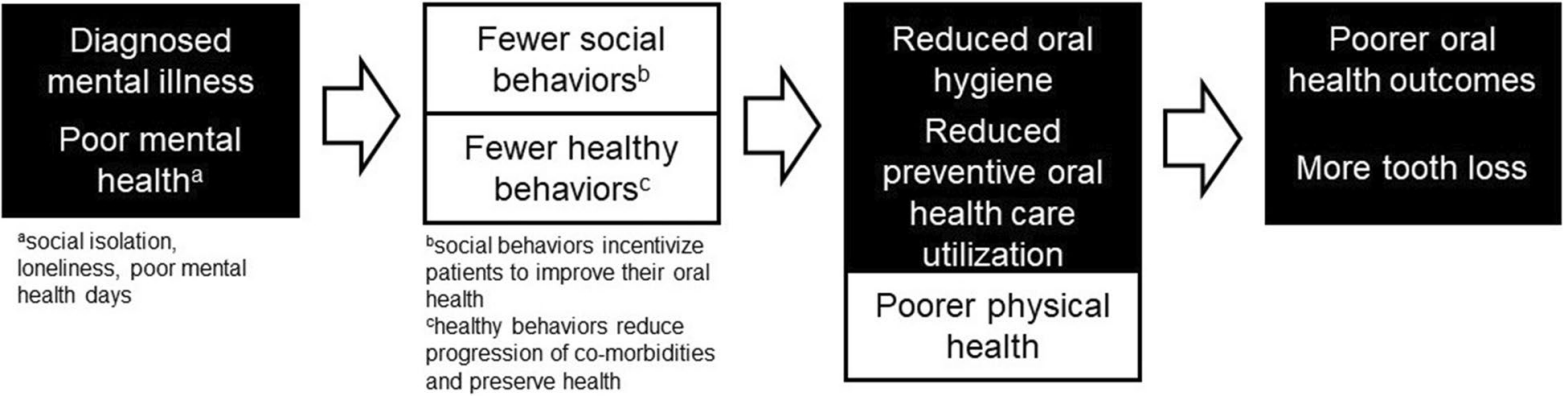
Proposed causal mechanism between poor mental health and poor oral health outcomes. Adapted from Qi et al. [8]

Considering these findings, Fig. 1 presents a potential explanation for the overall mechanism implicating poor MH as a cause of poor OH outcomes as demonstrated from cohort studies and the meta-analysis [7, 8].

As depicted in the theoretical model in Fig. 1, poor MH, whether resulting from a diagnosed MI or not, leads to fewer social behaviors that would incentivize the patient to present socially with good OH. It also leads to the patient engaging in fewer healthy behaviors, such as quitting tobacco smoking or adopting a healthier diet, which would maintain or improve their physical health. These healthy behaviors practiced at a lower rate by patients with poor MH include those aimed at improving or maintaining OH, such as engaging in oral hygiene and staying up-to-date with preventive dental visits. In addition, practicing fewer healthy behaviors leads to the progression of disease and poorer physical health. Lack of OH care along with increased physical co-morbidity both lead to poor OH outcomes, including increased tooth loss.

Although large epidemiologic analyses have been conducted on the association between poor MH and poor OH in several countries, recent studies have not focused on the US. The BRFSS is an annual anonymous cross-sectional survey done by phone in the US [15]. The BRFSS asks respondents to estimate the number of poor MH days in the last 30 days, and asks questions about both mental and physical co-morbidities, oral healthcare utilization and tooth loss. While these variables and the cross-sectional design may be limiting, the BRFSS provides a basis for a similar cross-sectional analysis as has been done before in datasets from other countries and at other time points [7]. The objective of this analysis was to test the hypothesis that among individuals in the 2020 BRFSS, after controlling for confounding, a trend showing that a larger number of poor MH days in the past month is associated with lower odds of oral health care utilization and higher odds of negative oral health outcomes.

## Methods

This is a cross-sectional analysis of the core dataset from the 2020 BRFSS, an annual health survey done anonymously by phone in the US that uses multi-stage sampling [15]. The exposure of interest was number of poor MH days in the past 30 days, and the OH outcomes of interest were most recent dental visit longer than one year ago including never (as a marker of lower utilization), and having lost six or more teeth including all (as a marker of poor OH). The details of the study design and analysis follow.

### Participants and setting

In 2020, a total of 401,958 participated in the BRFSS survey [15]. Records were removed if the respondent did not report when most recent dental visit took place (*n* = 4,667) or number of missing teeth (*n* = 9,117), as these were the OH outcomes of interest. Respondents were also removed if they failed to report number of poor MH days in the past 30 days (*n* = 7,009). To eliminate small cells in regression analysis, those who did not report their highest level of education (*n* = 1,434) or their general health status (*n* = 658) were removed, leaving 379,073 (94% of the initial dataset) remaining for analysis. As this dataset is anonymous and available for download from the internet, it is not considered human research under the Helsinki Declaration of 1964, and therefore ethical approval is not required for this analysis [16].

### Variables included

**Fig. 2:**
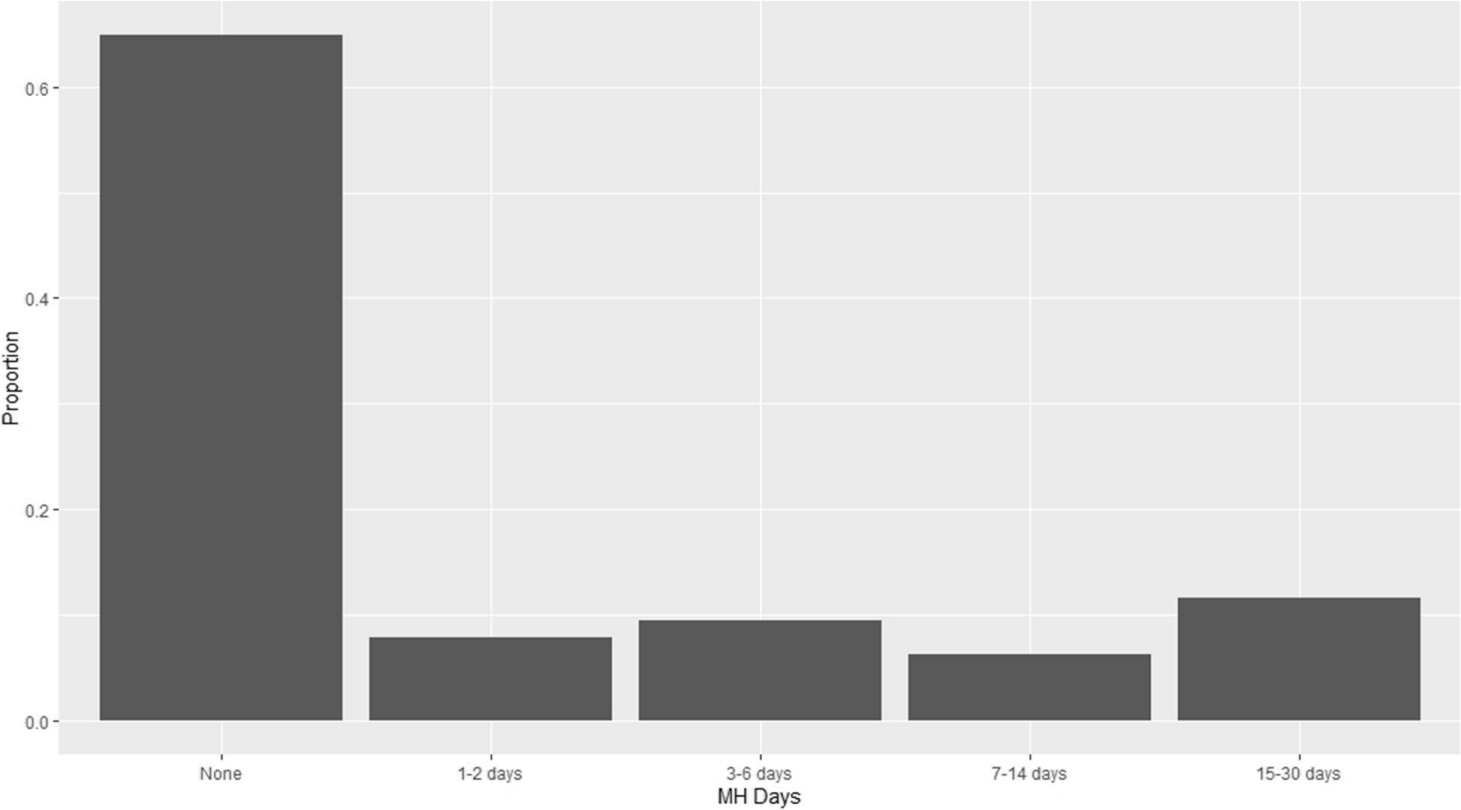
Relative frequency histogram of quartiles of mental health days in the 2020 Behavioral Risk Factor Surveillance Survey (BRFSS) dataset. Note: MH Days = number of mental health days in the past 30 days reported by respondent. To determine classifications of mental health (MH) days for this analysis, those answering “0” were placed in the reference group. Quartiles was chosen for the classification of the remainder because it enabled each stratum to have close to the same number of records, and because the classifications were somewhat intuitive, in that the first two categories included less than one week, the third category was one to two weeks, and the fourth category was more than two weeks

Table 1 provides a complete description of the variables used, as well as how they were recoded for analysis. In this cross-sectional analysis, the exposure was the answer to the question, “Now thinking about your mental health, which includes stress, depression, and problems with emotions, for how many days during the past 30 days was your mental health not good?”. To determine classifications of MH days for this analysis, those answering “0” were placed in the reference group, and the rest of the distribution was analyzed using the entire dataset (see Fig. 2).

**Table 1:** Variable coding

Role in Analysis	Question/ Topic	Variable Coding	Description
Eligibility and Exposure	Now thinking about your mental health, which includes stress, depression, and problems with emotions, for how many days during the past 30 days was your mental health not good?	• Number of days 0–30 • Don't know/ not sure/ refused	• Those answering 0 were placed in a "none" category • Those answering 1–30 were placed in categories based on quartiles of answers, where quartile 1 (Q1) was 1–2 days, Q2 was 3–6 days, Q3 was 7–14 days, and Q4 was 15–30 days • Unknowns were removed
Eligibility and Outcome	Including all types of dentists, such as orthodontists, oral surgeons, and all other dental specialists, as well as dental hygienists, how long has it been since you last visited a dentist or a dental clinic for any reason?	• Within the past year (anytime less than 12 months ago) • Within the past 2 years (1 year but less than 2 years ago) • Within the past 5 years (2 years but less than 5 years ago) • 5 or more years ago or never	• "Within the past year" considered up-to-date on dental care • Unknowns were removed
Eligibility and Outcome	Not including teeth lost for injury or orthodontics, how many of your permanent teeth have been removed because of tooth decay or gum disease?	• None • 1 to 5 • 6 or more (but not all) • All	• "6 or more (but not all)" and "all" combined as outcome for tooth loss • Unknowns were removed
Eligibility and Confounder	Are you male or female?	• Male • Female	• Must answer one of these two answers at beginning of survey to be eligible to continue • In regression analysis "male" (yes = 1) was used as an indicator variable
Confounder	BRFSS-derived age group variable	• 18 to 24 • 25 to 34 • 35 to 44 • 45 to 54 • 55 to 64 • 65 and older	No unknowns were present in native dataset In regression analysis, each level was modeled as an indicator variable with the lowest age group as reference
Confounder	BRFSS-derived Hispanic status variable	• Yes • No • Unknown	Indicator variable coded as Yes = 1 (with comparison "no" and "unknown") was used in regression analysis
Confounder	Recode of BRFSS-derived race variable	• White only • Black or African American only • American Indian or Alaskan native only • Asian only • Native Hawaiian or other Pacific Islander only • Other race • Unknown	"Other race" includes "other race only" and "multiracial" For regression analyses, "White only" and "unknown" were combined into the comparison group, and three indicator variables were developed: • "Black or African American only" (yes = 1) • "Asian only" (yes = 1), • any of the other races listed as an "other race" flag (yes = 1)
Confounder	Recode of BRFSS-derived marital status variable	• Coupled • Formerly married • Never married • Unknown	"Coupled" includes married and unmarried couples "Formerly married" includes divorced and widowed For regression, indicator variables were made for "never married" (yes = 1) and "formerly married" (yes = 1), with "coupled" and "unknown" combined in the comparison group
Eligibility and Confounder	What is the highest grade or year of school you completed?	• Less than high school graduate • High school graduate • Some college • College graduate	Unknowns were removed "Less than high school" includes respondents who never attended school, only attended kindergarten, or achieved grades up to 11 "High school graduate" includes grade 12 and GED "Some college" includes college or technical school one to three years "College graduate" includes college four years or more For regression analysis, a combined indicator variable for "less than high school graduate" and "high school graduate" (yes to either = 1) was created, and an indicator variable for "some college" (yes = 1), with the comparison group "college graduate"
Confounder	Is your annual household income from all sources:	• < $10,000 • $10,000 to < $15,000 • $15,000 to < $20,000 • $20,000 to < $25,000 • $25,000 to < $35,000 • $35,000 to < $50,000 • $50,000 to < $75,000 • $75,000 or more • Unknown	For regression analysis, "$75,000 or more" and "unknown" were combined for the comparison group, and indicator variables were made for each of the other levels where yes = 1 if the respondent reported that level
Confounder	Has a doctor, nurse, or other health professional ever told you that you had any of the following?	Asked about the following conditions: • Asthma • heart attack or myocardial infarction (MI) • angina or coronary heart disease (CHD) • stroke • skin cancer • other type of cancer • chronic obstructive pulmonary disease (COPD), emphysema, or chronic bronchitis • some form of arthritis, rheumatoid arthritis, gout, lupus, or fibromyalgia • depressive disorder • kidney disease • diabetes	For regression analysis, indicator variables were made for each reported co-morbidity where yes = 1, with all others in the comparison group
Eligibility and Confounder	Would you say that in general your health is–-	• Excellent • Very good • Good • Fair • Poor	Unknowns were removed For regression analysis, indicator variables were made for "fair" (yes = 1) and "poor" (yes = 1) with the comparison group including all other levels
Confounder	During the past 30 days, how many days per week or per month did you have at least one drink of any alcoholic beverage such as beer, wine, a malt beverage or liquor?	0 through 30. Those who answered 1 through 30 were classified as having an alcoholic drink within the last 30 days	For regression analysis, an indicator variable was made for having at least one alcoholic drink in the last 30 days (yes = 1), with all other records in the comparison group
Confounder	Have you smoked at least 100 cigarettes in your entire life? and Do you now smoke cigarettes every day, some days, or not at all?	To be classified as a current tobacco smoker, respondent must have said "yes" to smoking at least 100 cigarettes in their entire life, and either saying they currently smoke some days or every day	For regression analysis, current smoking status (1 = "yes") was used as an indicator variable, with all other records in the comparison group
Confounder	Do you currently use chewing tobacco, snuff, or snus every day, some days, or not at all?	• Yes • No	For regression analysis, an indicator variable for oral tobacco user (yes = 1) was created with all other levels as comparison group
Confounder	Do you have any kind of health care coverage, including health insurance, prepaid plans such as HMOs, or government plans such as Medicare, or Indian Health Service?	• Yes • No	For regression analysis, an indicator variable was made to indicate the lack of plan (not having plan = 1), with the comparison group reporting having a plan
Confounder	BRFSS-derived classification of body mass index (BMI)	• Underweight • Normal • Overweight • Obese • Unknown	For regression analysis, "normal" was combined with "unknown" for the comparison group, and indicator variables were developed for underweight (yes = 1), overweight (yes = 1), and obese (yes = 1)
Confounder	During the past month, other than your regular job, did you participate in any physical activities or exercises such as running, calisthenics, golf, gardening, or walking for exercise?	• Yes • No	For regression analysis, and indicator variable was made to indicate lack of exercise in the past 30 days (not exercising = 1), with those reporting exercise as the comparison group

As shown in Fig. 2, quartiles was the approach chosen for classification because it enabled each stratum to contain approximately the same number of records, and because the classifications were somewhat intuitive (e.g., the first two categories included less than one week in the last 30 days, the third category was one to two weeks, and the fourth category was more than two weeks). The specific class limits were: quartile 1 = 1–2 days, quartile 2 = 3–6 days, quartile 3 = 7–14 days, and quartile 4 = 15–30 days. In regression analysis, those answering 0 were placed in the reference group, and indicator variables were developed for each quartile and introduced into the models as independent variables (IVs).

Two dependent variables (DVs) were developed: one as a marker of OH utilization, and another as a marker of OH (see Table 1). The first DV involved classifying respondents by their answer to the question, “Including all types of dentists, such as orthodontists, oral surgeons, and all other dental specialists, as well as dental hygienists, how long has it been since you last visited a dentist or a dental clinic for any reason?”. Those answering “within the past year” were considered up-to-date, and the others (including “never”) were considered delayed; regression modeling predicted delayed dental visit (yes = 1, no = 0). The second DV involved the answer to the question, “Not including teeth lost for injury or orthodontics, how many of your permanent teeth have been removed because of tooth decay or gum disease?” Those answering “six or more (but not all)” or “all” were combined into a DV of “lost six or more teeth” (yes = 1, no = 0).

The other variables in the analysis were IVs to control for confounding. These included the demographic variables sex, age groups, Hispanic status, racial grouping, marital status, highest level of education, and income level (see Table 1 for coding). If respondent reported that “a doctor, nurse, or other health professional ever told” them they had the following co-morbidities, it was introduced as a control indicator IV in the analysis: asthma; history of heart attack or myocardial infarction (MI); angina or coronary heart disease (CHD); stroke, skin cancer; other type of cancer; chronic obstructive pulmonary disease (COPD), emphysema or chronic bronchitis; some form of arthritis, rheumatoid arthritis, gout, lupus, or fibromyalgia; depressive disorder; kidney disease; and diabetes. Other control variables included self-reported general health status, whether or not respondent had had at least one alcoholic drink in the past 30 days, whether or not respondent was a current tobacco smoker or user of oral tobacco, health insurance status, body mass index (BMI) classification, and whether or not respondent had engaged in physical activity in the past 30 days (see Table 1 for coding).

### Data analysis

First, descriptive analysis was conducted on the sample. Bivariate associations between the IVs and DVs were characterized with chi-square analysis, with α = 0.05. Next, to answer the research aims, two logistic regression models were developed – one to predict each binary DV (Model 1 DV: delayed dental visit: yes/no, and Model 2 DV: having lost six or more teeth: yes/no). As described earlier, a model-based (rather than a weight-based) approach was used, mainly because weight-based approaches unnecessarily increase the width of confidence intervals (CIs) [17, 18]. Nevertheless, to reduce controversy, both unweighted and weighted estimated odds ratios (ORs) and 95% CIs are presented [17, 18].

A manual stepwise selection process was used to determine IVs that were retained to control for confounding in final unweighted logistic regression models as control variables in addition to the IVs referring to the MH days exposure [19]. Control variables were retained in final unweighted models if their slopes were associated with a *p*-value of < 0.1. If *p*-values on slopes for indicator variables for the MH days exposure were statistically significant at α = 0.05 in final unweighted models, the null was considered to be rejected. Data analysis was conducted in R [20].

## Results

As described earlier, the original BRFSS 2020 dataset contained 401,958 rows, and after exclusions were applied, 379,073 records were available for analysis (94% of the original dataset). Table 2 presents bivariate associations between the MH and OH variables.

**Table 2:** Oral and mental health characteristics

			**Last Dental Visit**			**Tooth Loss**		
**Category**	**Level**	**All**	**More than 1 Year ago or never**	**Up to 1 Year ago**	**Chi-square *p*-value**	**Lost 6 or more teeth (including all)**	**Lost less than 6 teeth (including none)**	**Chi-square *p*-value**
		n, %	n, %	n, %	NA	n, %	n, %	NA
All	All	379,073, 100%	123,077, 32%	255,996, 68%		63,239, 17%	315,834, 83%	
How many of past 30 days that mental health was not good	None	244,782, 65%	74,717, 61%	170,065, 66%	*p* < 0.0001	40,790, 65%	203,992, 65%	*p* < 0.0001
	1–2 days	29,936, 8%	8,505, 7%	21,431, 8%		3,712, 6%	26,224, 8%	
	3–6 days	36,453, 10%	11,596, 9%	24,857, 10%		4,784, 8%	31,669, 10%	
	7–14 days	23,748, 6%	8,718, 7%	15,030, 6%		3,621, 6%	20,127, 6%	
	15–30 days	44,154, 12%	19,541, 16%	24,613, 10%		10,332, 16%	33,822, 11%	
Last dental visit	Within the past year	255,996, 68%	0, 0%	255,996, 100%	NC	29,383, 46%	226,613, 72%	*p* < 0.0001
	Between 1 and 2 years ago	47,234, 12%	47,234, 38%	0, 0%		8,633, 14%	38,601, 12%	
	Between 2 and 5 years ago	34,577, 9%	34,577, 28%	0, 0%		8,755, 14%	25,822, 8%	
	5 or more years ago or never	41,266, 11%	41,266, 34%	0, 0%		16,468, 26%	24,798, 8%	
Tooth loss status	0 teeth lost	203,019, 54%	55,888, 45%	147,131, 57%	*p* < 0.0001	0, 0%	203,019, 64%	NC
	1 to 5 teeth lost	112,815, 30%	33,333, 27%	79,482, 31%		0, 0%	112,815, 36%	
	6 or more teeth lost (but not all)	40,125, 11%	16,663, 14%	23,462, 9%		40,125, 63%	0, 0%	
	All teeth lost	23,114, 6%	17,193, 14%	5921, 2%		23,114, 37%	0, 0%	

Chi-square *p*-values considered statistically significant at α < 0.05. *NA* Not applicable, *NC* Not calculable

As shown in Table 2, about one third (32%) of the sample reported a most recent dental visit more than one year ago (or never), and 17% reported having lost 6 or more teeth. Also, almost two thirds of the sample (65%) reported having 0 poor MH days in the past 30 days; these individuals were significantly underrepresented among those with a delayed dental visit (*p* < 0.0001). On the other hand, those in the highest quartile of poor MH days (15–30 days among the past 30) were significantly overrepresented among those with a delayed dental visit (16% vs. 10% of those who are up-to-date, *p* < 0.0001) and those who had lost 6 or more teeth (16% vs. 11% of those who had lost five or fewer, *p* < 0.0001).

Table 3 provides a bivariate analysis of demographic characteristics and OH outcomes.

**Table 3:** Demographic and oral health characteristics

			**Last Dental Visit**			**Tooth Loss**		
**Category**	**Level**	**All**	**More than 1 Year ago or never**	**Up to 1 Year ago**	**Chi-square *p*-value**	**Lost 6 or more teeth (including all)**	**Lost less than 6 teeth (including none)**	**Chi-square *p*-value**
		n, %	n, %	n, %		n, %	n, %	
All	All	379,073, 100%	123,077, 32%	255,996, 68%	NA	63,239, 17%	315,834, 83%	NA
Sex	Male	173,826, 46%	61,779, 50%	112,047, 44%	*p* < 0.0001	28,097, 44%	145,729, 46%	*p* < 0.0001
	Female	205,247, 54%	61,298, 50%	143,949, 56%		35,142, 56%	170,105, 54%	
Age group	Age 18 to 24	24,578, 6%	8,060, 7%	16,518, 6%	*p* < 0.0001	151, 0%	24,427, 8%	*p* < 0.0001
	Age 25 to 34	42,699, 11%	17,079, 14%	25,620, 10%		1,368, 2%	41,331, 13%	
	Age 35 to 44	49,891, 13%	17,073, 14%	32,818, 13%		3,267, 5%	46,624, 15%	
	Age 45 to 54	58,789, 16%	18,773, 15%	40,016, 16%		6,506, 10%	52,283, 17%	
	Age 55 to 64	74,268, 20%	23,562, 19%	50,706, 20%		14,273, 23%	59,995, 19%	
	Age 65 or older	128,848, 34%	38,530, 31%	90,318, 35%		37,674, 60%	91,174, 29%	
Hispanic status	Hispanic	34,168, 9%	14,326, 12%	19,842, 8%	*p* < 0.0001	3,598, 3%	30,570, 12%	*p* < 0.0001
Race	White only	300,065, 79%	91,686, 74%	208,379, 81%	*p* < 0.0001	49,651, 79%	250,414, 79%	*p* < 0.0001
	Black or African American only	29,822, 8%	12,161, 10%	17,661, 7%		6,754, 11%	23,068, 7%	
	American Indian or Alaskan Native only	7,505, 2%	3,255, 3%	4,250, 2%		1,790, 3%	5,715, 2%	
	Asian only	9,820, 3%	3,055, 2%	6,765, 3%		641, 1%	9,179, 3%	
	Native Hawaiian or other Pacific Islander only	2,209, 1%	854, 1%	1,355, 1%		251, 0%	1,958, 1%	
	Other race or multi-racial	20,056, 5%	8,301, 7%	11,755, 5%		2,917, 5%	17,139, 5%	
	Unknown	9,596, 3%	3,765, 3%	5,831, 2%		1,235, 2%	8,361, 3%	
Marital status	Married or in unmarried couple	266,392, 70%	77,719, 63%	188,673, 74%	*p* < 0.0001	32,401, 51%	233,991, 74%	*p* < 0.0001
	Divorced or widowed	88,027, 23%	34,643, 28%	53,384, 21%		27,133, 43%	60,894, 19%	
	Never married	7,419, 2%	3,647, 3%	3,772, 1%		1,925, 3%	5,494, 2%	
	Unknown	17,235, 5%	7,068, 6%	10,167, 4%		1,780, 3%	15,455, 5%	
Highest level of education	Less than high school graduate	23,570, 6%	14,038, 11%	9,532, 4%	*p* < 0.0001	8,987, 14%	14,583, 5%	*p* < 0.0001
	High school graduate	99,726, 26%	41,885, 34%	57,841, 23%		24,711, 39%	75,015, 24%	
	Some college or technical school	105,761, 28%	35,509, 29%	70,252, 27%		18,425, 29%	87,336, 28%	
	College graduate	150,016, 40%	31,645, 26%	118,371, 46%		11,116, 18%	138,900, 44%	
Annual household income	< $10 k	11,849, 3%	6,657, 5%	5,192, 2%	*p* < 0.0001	3,934, 6%	7,915, 3%	*p* < 0.0001
	$10 k—< $15 k	12,642, 3%	7,276, 6%	5,366, 2%		5,084, 8%	7,558, 2%	
	$15 k—< $20 k	19,606, 5%	10,394, 8%	9,212, 4%		6,503, 10%	13,103, 4%	
	$20 k—< $25 k	26,043, 7%	12,396, 10%	13,647, 5%		7,515, 12%	18,528, 6%	
	$25 k—< $35 k	29,616, 8%	12,448, 10%	17,168, 7%		7,174, 11%	22,442, 7%	
	$35 k—< $50 k	41,914, 11%	14,566, 12%	27,348, 11%		7,524, 12%	34,390, 11%	
	$50 k—< $75 k	50,717, 13%	14,061, 11%	36,656, 14%		6,226, 10%	44,491, 14%	
	$75 k or more	115,967, 31%	21,955, 18%	94,012, 37%		6,919, 11%	109,048, 35%	
	Unknown	64,100, 17%	21,087, 17%	43,013, 17%		11,417, 18%	52,683, 17%	

Chi-square *p*-values considered statistically significant at α < 0.05. *NA* Not applicable

As seen in Table 3, in bivariate analysis, the strongest associations between demographic characteristics and OH variables were seen with age group, race, marital status, and income. Age group showed a strong, significant direct dose–response trend association with lost teeth, in that while individuals aged 65 and older only made up 34% of the sample, they represented 60% of the respondents who had lost 6 or more teeth (*p* < 0.0001). Although 79% of the sample was White, Whites made up only 74% of those with delayed dental visit (*p* < 0.0001). Being in a married or unmarried couple and being of higher education and/or income were associated with better OH outcomes. While 70% of the sample were married or in an unmarried couple, they made up only 63% of those with a delayed dental visit, and only 51% of those having lost 6 or more teeth (*p* < 0.0001). Forty-percent of the sample reported having graduated from college, but these respondents made up only 26% of those with a delayed dental visit, and 18% of those who had lost six or more teeth (*p* < 0.0001). Almost one third (31%) of the sample was in the highest income group, but they made up only 17% of those with a delayed dental visit (*p* < 0.0001), and 18% of those having lost six or more teeth (*p* < 0.0001).

Table 4 presents a bivariate analysis of clinical characteristics and OH outcomes.

**Table 4:** Clinical and oral health characteristics

			**Last Dental Visit**			**Tooth Loss**		
**Category**	**Level**	**All**	**More than 1 Year ago or never**	**Up to 1 Year ago**	**Chi-square *p*-value**	**Lost 6 or more teeth (including all)**	**Lost less than 6 teeth (including none)**	**Chi-square *p*-value**
		n, %	n, %	n, %	NA	n, %	n, %	NA
All	All	379,073, 100%	123,077, 32%	255,996, 68%		63,239, 17%	315,834, 83%	
Co-morbidities reported	Current asthma	35,800, 9%	12,992, 11%	22,808, 9%	*p* < 0.0001	8,259, 13%	27,541, 9%	*p* < 0.0001
	Heart attack	20,209, 5%	9,000, 7%	11,209, 4%	*p* < 0.0001	8,519, 13%	11,690, 4%	*p* < 0.0001
	Coronary heart disease	20,900, 6%	8,367, 7%	12,533, 5%	*p* < 0.0001	8,043, 13%	12,857, 4%	*p* < 0.0001
	Stroke	14,266, 4%	6,367, 5%	7,899, 3%	*p* < 0.0001	5,768, 9%	8,498, 3%	*p* < 0.0001
	Chronic obstructive pulmonary disease	28,923, 8%	14,094, 11%	14,829, 6%	*p* < 0.0001	13,733, 22%	15,190, 5%	*p* < 0.0001
	Arthritis	116,068, 31%	39,802, 32%	76,266, 30%	*p* < 0.0001	33,364, 53%	82,704, 26%	*p* < 0.0001
	Kidney disease	13,885, 4%	5,461, 4%	8,424, 3%	*p* < 0.0001	4,909, 8%	8,976, 3%	*p* < 0.0001
	Depression	71,327, 19%	28,094, 23%	43,233, 17%	*p* < 0.0001	16,283, 26%	55,044, 17%	*p* < 0.0001
	Diabetes	48,233, 13%	19,105, 16%	29,128, 11%	*p* < 0.0001	15,802, 25%	32,431, 10%	*p* < 0.0001
	Skin cancer	34,213, 9%	8,201, 7%	26,012, 10%	*p* < 0.0001	7,654, 12%	26,559, 8%	*p* < 0.0001
	Other cancer	34,242, 9%	10,572, 9%	23,670, 9%	*p* < 0.0001	9,513, 15%	24,729, 8%	*p* < 0.0001
Self-reported general health	Excellent	78,485, 21%	19,193, 16%	59,292, 23%	*p* < 0.0001	5,215, 8%	73,270, 23%	*p* < 0.0001
	Very Good	132,216, 35%	35,769, 29%	96,447, 38%		14,661, 23%	117,555, 37%	
	Good	111,808, 29%	40,337, 33%	71,471, 28%		21,991, 35%	89,817, 28%	
	Fair	42,599, 11%	20,023, 16%	22,576, 9%		14,676, 23%	27,923, 9%	
	Poor	13,965, 4%	7,755, 6%	6,210, 2%		6,696, 11%	7,269, 2%	
Alcohol use	At least one drink in past 30 days	184,278, 49%	52,797, 43%	131,481, 51%	*p* < 0.0001	20,571, 33%	163,707, 52%	*p* < 0.0001
	No drinks in past 30 days	171,178, 45%	62,261, 51%	108,917, 43%		39,067, 62%	132,111, 42%	
	Unknown	23,617, 6%	8,019, 7%	15,598, 6%		3,601, 6%	20,016, 6%	
Current tobacco use	Current smoker	49,294, 13%	26,155, 21%	23,139, 9%	*p* < 0.0001	16,188, 26%	33,106, 10%	*p* < 0.0001
	Current oral tobacco use	12,258, 3%	6,013, 5%	6,245, 2%	*p* < 0.0001	2,168, 3%	10,090, 3%	*p* < 0.0001
Current health insurance	Yes	345,820, 91%	910, 1%	241,691, 94%	*p* < 0.0001	1,367, 2%	287,502, 91%	*p* < 0.0001
Body mass index category	Underweight	5,559, 1%	2,092, 2%	3,467, 1%	*p* < 0.0001	1,243, 2%	4,316, 1%	*p* < 0.0001
	Normal	104,495, 28%	30,944, 25%	73,551, 29%		15,366, 24%	89,129, 28%	
	Overweight	122,725, 32%	37,701, 31%	85,024, 33%		19,924, 32%	102,801, 33%	
	Obese	109,926, 29%	40,573, 33%	69,353, 27%		21,821, 35%	88,105, 28%	
	Unknown	36,368, 10%	11,767, 10%	24,601, 10%		4,885, 8%	31,483, 10%	
Exercise within the last 30 days	Yes	291,135, 77%	243, 0%	207,433, 81%	*p* < 0.0001	315, 0%	253,289, 80%	*p* < 0.0001

Chi-square *p*-values considered statistically significant at α < 0.05. *NA* Not applicable

As shown in Table 4, strong associations are seen between certain co-morbidities, self-reported general health, alcohol use, tobacco smoking, and regular exercise. The most prevalent co-morbidity in the sample was arthritis (31%), followed by depression (19%) and diabetes (13%). Those with arthritis were significantly overrepresented among those who had lost 6 or more teeth (53%, *p* < 0.0001). Those with depression were also significantly overrepresented among those with a delayed dental visit (23%, *p* < 0.0001) and those who had lost six or more teeth (26%, *p* < 0.0001), but to a smaller degree. A significant inverse dose–response trend was identified with general health, in that one-fifth of the sample (21%) reported their general health was excellent, but these individuals only made up 16% of those with a delayed dental visit and 8% of those who had lost six or more teeth (*p* < 0.0001 for both analyses). Almost half (49%) of the sample reported using alcohol within the past 30 days, and these individuals were significantly underrepresented for both OH outcomes (delayed dental visit 43%, lost six or more teeth 33%, *p* < 0.0001 for both). While only 13% of the sample were tobacco smokers, they were significantly overrepresented among those with a delayed dental visit (21%, *p* < 0.0001) and those who had lost 6 or more teeth (26%, *p* < 0.0001). Most of the sample (91%) had health (but not necessarily dental) insurance [5], and over three-fourths (77%) of the sample exercised within the past 30 days. Not having health insurance and not exercising in the past 30 days was strongly and significantly associated with both outcomes (*p* < 0.0001).

**Fig. 3:**
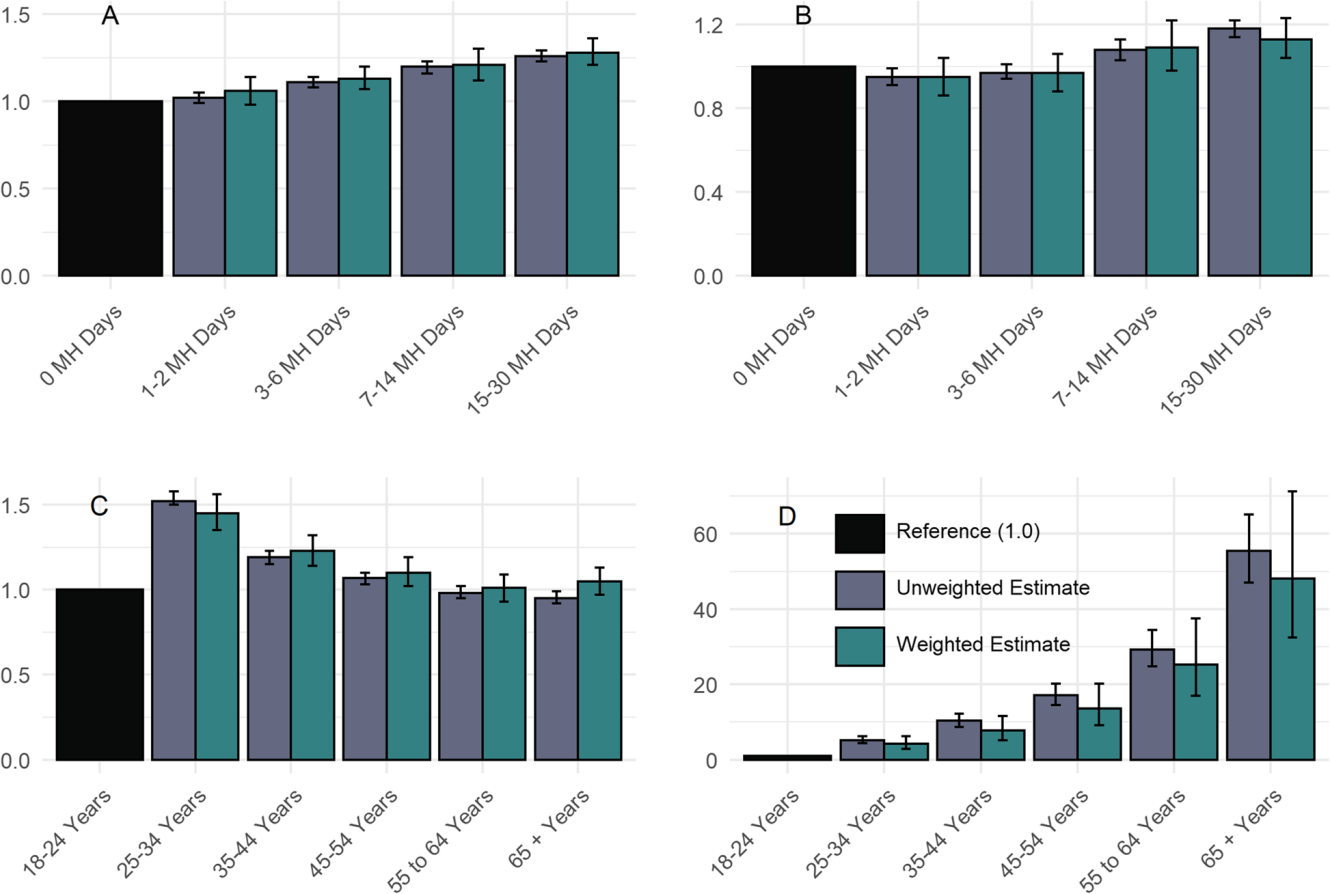
Odds ratios and 95% confidence intervals from unweighted and weighted logistic regression models for mental health days and age groups. Figure 3A shows the odds ratios (ORs) and 95% confidence intervals (CIs) for the estimates for mental health (MH) days in the past 30 days from Model 1, where the dependent variable (DV) was most recent dental visit longer than one year ago. Figure 3B shows the ORS and 95% CIs for the estimates for MH days from Model 2. Figures 3A and B show that while there is a clear direct dose–response relationship between number of MH days and odds of delayed dental visit, the relationship between number of MH days and having lost six or more teeth is not straightforward. The association is not as strong, and only is significant in the top two quartiles of MH days. By contrast, as shown in Figs. 3C and D, age had a strong inverse dose–response relationship on odds of delayed dental visit from Model 1, while it had an extremely strong direct dose–response relationship with odds of having lost six or more teeth, with the magnitude of association increasing by over tenfold for the top two age groups

Table 5 provides the results from weighted and unweighted adjusted logistic regression models, and Fig. 3 provides visualizations from the ORs and 95% CIs for the poor MH days and age group indicator variables from these models to provide a visual comparison.

**Table 5:** Regression model results

**Category**	**Variable**	**Odds ratio (95% confidence interval)**			
		**Model 1** **Outcome: Delayed Dental Visit**		**Model 2** **Outcome: Loss of 6 + Teeth**	
		**Unweighted**	**Weighted**	**Unweighted**	**Weighted**
Mental health days	0 of the last 30 days where mental health was not good	Reference	Reference	Reference	Reference
	1–2 of the last 30 days where mental health was not good	1.02 (0.99–1.05)	1.06 (0.98–1.14)	0.95 (0.91–0.99)*	0.95 (0.86–1.04)
	3–6 of the last 30 days where mental health was not good	1.11 (1.08–1.14)*	1.13 (1.07–1.20)*	0.97 (0.94–1.01)	0.97 (0.88–1.06)
	7–14 of the last 30 days where mental health was not good	1.20 (1.16–1.23)*	1.21 (1.12–1.30)*	1.08 (1.03–1.13)*	1.09 (0.98–1.22)
	15–30 of the last 30 days where mental health was not good	1.26 (1.23–1.29)*	1.28 (1.21–1.36)*	1.18 (1.14–1.22)*	1.13 (1.04–1.23)*
Age category	Age 18 to 24	Reference	Reference	Reference	Reference
	Age 25 to 34	1.52 (1.50–1.58)*	1.45 (1.35–1.56)*	5.21 (4.39–6.17)*	4.19 (2.79–6.27)*
	Age 35 to 44	1.19 (1.15–1.23)*	1.23 (1.14–1.32)*	10.31 (8.74–12.16)*	7.72 (5.17–11.53)*
	Age 45 to 54	1.07 (1.03–1.10)*	1.10 (1.02–1.19)*	17.08 (14.51–20.12)*	13.51 (9.07–20.12)*
	Age 55 to 64	0.98 (0.95–1.02)	1.01 (0.93–1.09)	29.22 (24.83–34.38)*	25.22 (16.97–37.47)*
	Age 65 or older	0.95 (0.92–0.99)*	1.05 (0.97–1.13)	55.39 (47.08–65.18)*	48.09 (32.43–71.31)*

* statistically significant at α <0.05. Logistic regression modeled probability of the delayed dental visit (longer than 1 year), and the probability of having lost 6 or more teeth. Additional control variables considered to be included in both models were the following: ethnicity, race, marital status, highest level of education, household income level, status of the following health conditions: asthma, history of heart attack, coronary heart disease, stroke, chronic obstructive pulmonary disease, arthritis, kidney disease, depression, diabetes, skin cancer, and other cancer, general health level (fair and poor levels), alcohol use within the last 30 days, current tobacco smoker, current oral tobacco user, lack of health insurance, obesity status, and lack of exercise. All control variables retained in final models were statistically significant at α < 0.1

As shown in Table 5 and Fig. 3, in both weighted and unweighted adjusted logistic regression models, there was a significant, direct dose–response trend in that presence in higher strata of MH Days was associated with higher odds of both outcomes. In terms of Model 1, where the DV was delayed dental visit, after controlling for confounding, those in the second quartile of poor MH days (3 to 6 days in the past 30) had 11% higher odds of the outcome compared to those with 0 poor MH days (OR 1.11, 95% CI 1.08–1.14), and those in the highest quartile (15 to 30 poor MH days in the past 30) had 26% higher odds of the outcome compared to those 0 poor MH days (OR 1.26, 95% CI 1.23–1.29).

With the second outcome of having lost six or more teeth as shown in Model 2, after controlling for confounding, only the top two quartiles were associated with significantly higher odds compared to those with 0 poor MH days in the past 30. With respect to unweighted estimates, those in the third quartile of poor MH days (7 to 14 in the past 30) had 8% higher odds of having lost 6 or more teeth (OR 1.08, 95% CI 1.03–1.13), and those in the fourth quartile had 18% higher odds of the outcome (OR 1.18, 95% CI 1.14–1.22) compared to the reference group.

Also reported in Table 5 and on Fig. 2 are the estimates for the age groups (compared to the youngest age group, age 18 to 24 years, as the reference) from both models. These are presented to compare age and poor MH days in terms of magnitude and direction of association with the two DVs. The oldest age group had significantly lower odds of the outcome of delayed dental visit than the reference group (unweighted OR 0.95, 95% CI 0.92–0.99), and the trend in relationship between age strata and odds of the outcome were reverse dose–response. On the other hand, there was a strong direct dose–response trend between higher age strata and of having lost 6 or more teeth, with the unweighted OR doubling from the second age group (5.21, 95% CI 4.39–6.17) to the third lowest age group (17.08, 95% CI 14.51–20.12), and other ORs increasing by over ten-fold for each subsequent age group.

A post-hoc descriptive analysis was done to characterize those in the highest quartile of poor MH days as compared to the full sample (data not shown). Those in the highest quartile of poor MH days were more likely than the overall sample to be female, younger, divorced or widowed, a tobacco smoker, and obese. They were also more likely to report poorer general health, and to have asthma, COPD, and arthritis, while only 57% reported being diagnosed with depression.

## Conclusions

After controlling for other variables (including other physical and MH co-morbidities), this cross-sectional analysis found a dose–response trend, in that higher classifications of MH days in the past month (as a measure of HRQOL) were associated with higher odds of most recent dental visit being delayed past one year among a large, representative sample of US residents. Such a strong association was not seen with the other outcome of having lost six or more teeth, where those with at least one week of poor MH days in the past month had higher odds of having lost six or more teeth compared to those with no poor MH days in the past month. Although a cross-sectional association between age group and delayed dental visit was also found, the magnitudes of association were small. Finally, an extremely strong direct dose–response trend was found in the association between age group and the odds of having lost 6 or more lost teeth. As it is already known that elders are at much higher risk for tooth loss, this finding emphasizes the importance of regular OH utilization and the mitigation of other risk factors for tooth loss in all age groups in the US, but especially older ones [21].

As stated earlier, cross-sectional studies of MH and OH outcomes typically hypothesize the direction of causation depicted in Fig. 1 [7]. This research sought to study the association between poor MH days (as a measure of poor HRQOL) above and beyond any assigned diagnosis, given the lack of access in the US healthcare system. It has already been well-established that OH issues are more prevalent in patients with severe mental illness (SMI), meaning those who experience serious functional impairment due to a diagnosed mental illness (MI) [12]. The causes behind such a high risk in this population include both behavioral risk factors such as high sugar intake, tobacco smoking, and alcohol consumption, as well as other risk factors, such as dry mouth due to medication, lack of motivation for maintaining oral health, negative attitudes towards and anxiety about dental care, and cost barriers [12]. One systematic review found that patients with SMI had 2.8 times the odds of being edentulous [22].

While evidence exists to support the model in Fig. 1, evidence also supports tooth loss as being a potential cause of cognitive impairment [10, 23]. In this mechanism, there are two main causal factors [10]. First, increased tooth loss leads the patient to have difficulty eating, and they are forced to adopt a less healthy diet, and second, the increased tooth loss leads to an increased total body inflammatory load [10]. Based on this hypothesis, an analysis of data from the CLHLS found that among patients without dentures, any tooth loss was associated with incident cognitive impairment, but there was not a dose–response relationship [10]. Other evidence includes a cross-sectional study of a sample of Japanese elders, which found that score on the Mini-Mental State Examination (MMSE) was significantly associated with number of lost teeth [23]. While it may be the case that poor mental or neurological health is caused by increased lost teeth, the strength of association found in studies has been weak, and it is unlikely that this explains the link between poor MH and poor OH at the population level.

The results of the current analysis agree with the literature in terms of the basic premise that poor MH provides obstacles to OH care which can lead to poor OH outcomes. However, this study highlights particular challenges seen in the US. First, only 57% of those reporting over two weeks of poor MH days in the past month were actually diagnosed with depression, which likely reflects barriers to MH care in the US healthcare system. It has been estimated that one in four US adults who meet the diagnostic criteria for a MI had an unmet need for MH treatment in the past year, most commonly due to cost, and this unmet need has been found to be higher in the low-income, uninsured, and non-White [11, 24]. Second, by including physical health, MH, and OH variables in one analysis, the connection between poor physical health, poor MH, and poor OH became especially evident. As a specific example, in other research, those with arthritis report difficulty maintaining their OH due to the pain and extra time associated with oral self-care, so they are at a higher risk of tooth loss, as was reflected in this analysis [25, 26]. Further, arthritis has been linked to depression in US adults [27].

Third, the age consideration in the analysis can also be linked to nuances in access to care in the US, as individuals aged 65 and over are almost universally on the public health insurance program, Medicare [5]. Medicare fosters access to the physical healthcare system, but does not routinely provide dental insurance or access to OH care [5]. Additionally, the Affordable Care Act (ACA) passed in 2012 in the US facilitated almost universal health plan coverage for individuals under age 65, so while over 90% of the sample reported being on a health plan, they were probably still encountering barriers to accessing OH care [5, 11]. And even with access to physical healthcare, there are still access barriers to MH care due to lack of MH providers and facilities to deliver care in the US [28, 29] as well as extra costs associated with accessing MH care [11].

Ultimately, the results of this analysis say less about the direct connection between MH and OH, and more about the challenges individuals in the US face as they age and accrue physical, MH, and OH co-morbidities. Not being able to access physical care in the US due to barriers such as cost or lack of insurance places a vulnerable section of society at risk for advancing physical, mental, and oral disease. Those who are able to access care through Medicare or health plans facilitated through the ACA for their physical conditions, such as arthritis, may still suffer advancing MH and OH conditions due to barriers in accessing both MH and OH care. As advancing age is an immutable risk factor strongly associated with tooth loss, the results of this analysis highlight the importance of removing barriers to both MH and OH care in older groups in the US – those in the Medicare age bracket as well as those advancing toward the age of Medicare eligibility.

While the BRFSS dataset is nationally-representative and known for its high quality, there are still limitations to this analysis. The cross-sectional nature prevents determinations of causality, not all confounders were measured and were able to be placed in the model, disputes continue about the use of weights in regression analysis of BRFSS data, and misclassification of disease status is apparent in how the questions about diagnosed co-morbidities are asked. The lack of clinical parameters such as the Decayed, Missing due to caries, and Filled Teeth (DMFT) Index reduce the clarity of the measurement of OH outcomes. Further, it is not clear what exactly “poor MH days” means in a clinical sense, so addressing this as an unwanted population-level exposure is difficult to conceive of from a policy standpoint. Finally, although the estimates found in this study for poor MH days were small in magnitude, they were derived from an extremely large dataset and likely represent an additional issue to consider when addressing the prevalence of poor OH health in the US.

In conclusion, after controlling for confounding, those reporting more MH days in in the past month had a significantly higher odds of delaying their dental visit longer than a year, and having lost six or more teeth, in this cross-sectional analysis of a representative sample of US adults. However, this finding says less about the connection between poor MH and poor OH, and more about barriers to accessing care in the US, where most health plans do not cover oral healthcare, and where serious obstacles to accessing MH services remain. As has been recommended by others, Medicare and other health plans in the US should consider including dental coverage [5], and the US needs to continue to work towards removing barriers to accessing MH services [30]. The findings also suggest that even though there is a high rate of health insurance plan coverage in the US due to polices enacted over the past decade, patients with chronic conditions who face barriers to accessing OH and MH services will continue to be at unnecessarily high risk for disease progression.

## Data Availability

BRFSS data are available online here: https://www.cdc.gov/brfss/data_documentation/index.htm. The 2020 core dataset was the only dataset used in the analysis. Public access to these surveillance datasets is open. No administrative permissions are required to access the core datasets posted to the BRFSS portal for download. A “frequently asked questions” page that clarifies the use of public BRFSS datasets is accessible here: https://www.cdc.gov/brfss/about/brfss_faq.htm. R code for the analysis is available upon request to the author.

## Abbreviations

ACA: Affordable Care Act
BMI: Body mass index
BRFSS: Behavioral Risk Factor Surveillance System
CHD: Coronary heart disease
CI: Confidence interval
CLHLS: Chinese Longitudinal Healthy Longevity Survey
COPD: Chronic obstructive pulmonary disease
DMFT: Decayed, Missing due to caries, and Fill Teeth
DV: Dependent variable
ELSA: English Longitudinal Study of Ageing
HRQOL: Health-related quality-of-life
IV: Independent variable
MH: Mental health
MI: Mental illness
MMSE: Mini-Mental State Examination
OH: Oral health
OIDP: Oral Impacts on Daily Performances
OR: Odds ratio
SMI: Severe mental illness
US: United States
